# Treatment of Advanced Gingival Recession Secondary to Surgical Failure with Large Size Deepithelized Gingival Graft Associated with a Modified Tunnel Flap

**DOI:** 10.1155/2023/8954257

**Published:** 2023-09-19

**Authors:** Lingzhou Zhao, Yuan Gao, Yihan Xiao, Xinwei Tan, Xiaochen Li, Tianzheng Deng

**Affiliations:** Department of Stomatology, Air Force Medical Center, The Fourth Military Medical University, Beijing 100142, China

## Abstract

**Objective:**

To describe the use of a large size deepithelized gingival graft (DGG) associated with full-split tunnel technique in a clinical case of advanced gingival recession secondary to surgical failure (GRSF). *Clinical Considerations*. The presented case report helped to achieve satisfactory root coverage, ideal keratinized tissue gain, improvement in soft tissue quality and esthetics, scar deformity correction, and vestibular depth deepening with a one-step procedure of large size DGG associated with full-split tunnel technique for a condition of deep gingival recessions of 7-11 mm caused by a failed bone implantation surgery.

**Conclusions:**

The large size DGG associated with full-split tunnel technique provided a versatile one-step procedure to obtain ideal results for advanced GRSF. *Clinical Significance*. GRSF that is generally associated with inadequate keratinized tissue and scar formation could be rather difficult to deal with. The large size DGG associated with full-split tunnel technique, as a one-step procedure, provided a predictable and practical treatment modality.

## 1. Introduction

Gingival recessions are the exposure of tooth root as the apical displacement of the gingival margin relative to cemento-enamel junction (CEJ) [1]. The causes of gingival recession are associated with many factors such as thin periodontal phenotype, alveolar bone dehiscence, tooth malposition, muscular pull, periodontal disease, and trauma from occlusion or incorrect tooth brushing [1]. Moreover, gingival recessions can also arise from iatrogenic factors such as surgical failure, which are often associated with relatively large amount of soft tissue missing, scar deformity formation and poor soft tissue quality, decreased vascularization, and severely impaired esthetics and function. The treatment for such gingival recession secondary to surgical failure (GRSF) could be rather difficult, and hitherto, the report on the treatment of GRSF is scarce.

The reported surgical techniques for gingival recession defect repair can be divided into the pedicle flap procedures [2–4] and the soft tissue/substitute grafting techniques [5, 6]. The pedicle flap procedures can be excluded from the treatment of GRSF considering the poor soft tissue quality and scar formation of GRSF. The techniques of connective tissue graft (CTG) associated with coronal advanced flap (CAF) or tunnel flap have been demonstrated with ideal root coverage in terms of the regular gingival recession defects [7]. Especially, the tunnel flap owns the advantage of minimal blood supply impairment that can lead to ideal root coverage, esthetics, gain of keratinized tissue width and thickness, and vestibular depth maintenance when associated with CTG [8]. Hence, it is proposed by us that with suitable modification, the tunnel flap associated with CTG technique may be effective to treat GRSF.

Subepithelial CTG harvesting techniques, such as the trap-door and single-incision approaches, are widely recommended to achieve a primary intension palatal wound healing to reduce the postoperative morbidity [9]. However, these techniques require a certain amount of palatal thickness to avoid desquamation of the undermined superficial flap due to low thickness with compromised vascularization. Indeed, a common complication of these harvesting techniques is dehiscence/necrosis of the primary flap [10]. To avoid the complication, a certain amount of the subepithelial connective tissue should be left to the primary flap, and consequently, the deeper connective tissue harvested is less dense, less stable, richer in fatty and glandular tissue, and more prone to shrinkage with poorer root coverage effect [10, 11]. In addition, it also shows the disadvantage of limited donor tissue in case of relatively lesser palatal tissue thickness [10]. On contrary, deepithelized gingival graft (DGG) approach may be more favorable, especially for the lesser palatal thickness situations. With the DGG approach, it is much easier to obtain adequate thickness and size of connective tissue graft and simultaneously a residual thickness of soft tissue to cover the palatal bone. After the removal of the epithelium, the most superficial lamina propria can be kept in the graft, which is denser, firmer, and richer in fibrous connective tissue [11, 12], leading to decreased shrinkage of the grafts and better root coverage effect [13]. The supposed less favorable and more painful postoperative course from the secondary intention palatal wound healing pattern of the DGG approach [14] was not confirmed, but instead, comparable postoperative patient-centered outcomes including pain, discomfort, and bleeding for DGG (with the palatal wound being protected by collagen matrix) and subepithelial CTG harvesting procedures were reported [10, 15].

Given the extensive scar deformity of GRSF, tension-free coronal advancement of the tunnel flap to fully cover the graft and the exposed root surface is rather difficult. The exposed part of graft will have quite a poor early blood supply being inclined to shrinkage. Hence, a large and thick DGG may be used to compensate the poor blood supply and graft shrinkage. The present report proposes such a large size DGG associated with tunnel flap as a one-step surgery for treating GRSF. Excellent root coverage, soft tissue quality improvement, scar tissue deformity correction, and increase in keratinized tissue width and thickness are achieved.

## 2. Case Report

**Figure 1:**
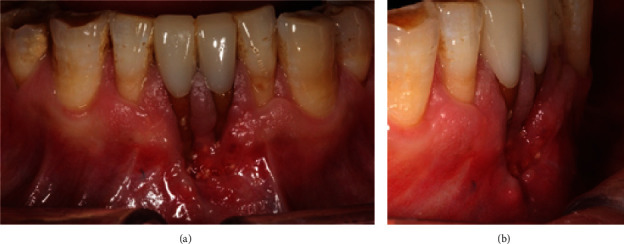
Front view (a) and lateral view (b) of the deep gingival recessions on the mandibular central incisors.

A 53-year-old nonsmoking male patient presented for repairing the gingival recessions on the mandibular central incisors, caused by a failed bone graft surgery conducted two weeks before. The initial intraoral picture (Figure 1) showed that the two mandibular central incisors had crowns. With the crown margin as the reference, the left mandibular central incisor had a gingival recession of 7 mm in depth, and the right one showed a gingival recession of 11 mm, measured with a periodontal probe (Hu-Friedy, USA). The exposed root surfaces showed discoloration. The soft tissue apical to the recessions was tender and fragile with immature epithelialization, and many bone graft granules were seen in the soft tissue. The left and right lateral incisors also had gingival recessions of 3 mm each, measured with a periodontal probe (Hu-Friedy, USA). The defects were diagnosed as GRSF. The patient showed a generally thick periodontal phenotype.

**Figure 2:**
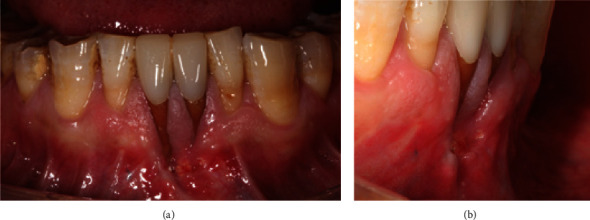
Front view (a) and lateral view (b) of the deep gingival recessions in the mandibular central incisors 50 days after initial presentation and immediately before surgery.

The wound was allowed to heal for another 50 days before implementing the recession repair surgery (Figure 2). Compared with the initial condition, a much more mature soft tissue condition was achieved, and the depth of the gingival recessions decreased slightly. The gingival recessions for the left and right central incisors are 6 and 11 mm, respectively. There was minimal keratinized tissue apical to the recession of the left mandibular central incisor, while the keratinized tissue for the right mandibular central incisor was still absent. Dense scar deformities with embedded bone graft granules apical to the gingival recessions were observed. The patients showed no gingival inflammation or bleeding on probing around the mandibular anterior teeth.

**Figure 3:**
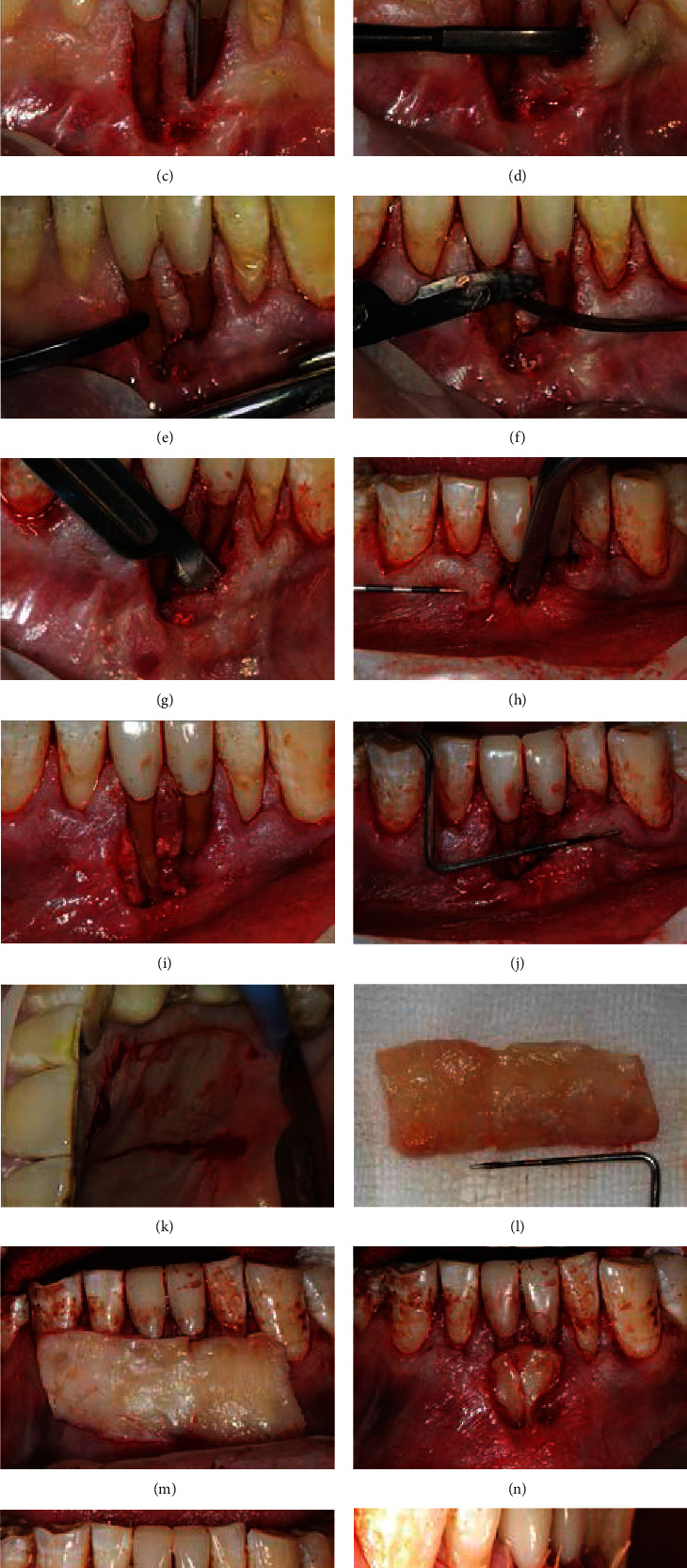
The step-by-step preparation of the tunnel flap for 33-43 (a–i), the harvest of DGG (j–l), and the insertion of DGG into the tunnel flap and suturing (m–p).

The patient was instructed to irrigate with 0.2% chlorhexidine digluconate (Colgate, USA). After application of local anesthetic with 4% articaine hydrochloride (Primacaine, France), mechanical treatment of the denuded root surface was conducted with the curette (Younger-Good 7/8, Hu-Friedy, USA) (Figures 3(a) and 3(b)). For the deep recessions on the two mandibular central incisors, intrasulcular incisions were introduced carefully to the bone surface sparing the papilla area (Figure 3(c)). With the gingival recession area as the access, a full-thickness mucoperiosteal separation laterally and apically to beyond the MGJ was performed on the facial aspect of the mandibular left canine to right canine with a microsurgical elevator (PPA-ELA, Hu-Friedy, USA) (Figure 3(d)). Specially, care was taken to elevate the gingival band between the two mandibular central incisors in full thickness, but unfortunately, it teared off easily at the apical part (Figure 3(e)). The gingival band was then cautiously deepithelized to be kept under the graft later (Figure 3(f)). Papilla elevation has been suggested by many periodontists in the tunnel technique for tension-free coronal advancement of the flap [16–18], while it was not employed in this case since our clinical experience shows that such procedure frequently leads to undesirable papilla recession and black triangle formation. Afterwards, the tunnel dissection was further extended apically in partial-thickness with a 15c blade (Medicon, Germany) (Figure 3(g)) and a Modified Orban Knife (Hu-Friedy, USA). The partial-thickness flap was conducted consecutively from one side of the tunnel flap to the other side to form a completely interconnected underlying space for an easy insertion of the graft. With the relative deep flap detachment apically and the continuous severing of the periosteum when commencing partial-thickness flap detachment, the tunnel flap could be coronally advanced to a great extent without tension (Figure 3(h)). The prepared accepting site was shown in Figure 3(i), and the length of the DGG graft needed was estimated to be 30 mm (Figure 3(j)). A free gingival graft (FGG) of 30 × 10 mm^2^ in size and about 2-2.5 mm in thickness was harvested from the left palate (Figure 3(k)). The palatal wound was protected with a collagen matrix, which was maintained in situ with 5-0 continuous sutures. The graft was carefully deepithelialized with a new 15c blade to form DGG (Figure 3(l)). DGG was then inserted through the gingival recession site on tooth 31 and 41 into the tunnel flap easily, aligned facial to teeth 33-43, and positioned to the crown margin levels (Figures 3(m) and 3(n)). The overlying tissues were coronally positioned as far as possible under the premise of no much tension and stabilized by 5-0 interrupted sling sutures and compressive outer mattress sutures (Vicryl Rapide, Johnson & Johnson, USA). A great part of the graft, about 9 mm in height, was exposed due to the difficulty in tension release (Figures 3(o) and 3(p)). No periodontal dressing was administrated. The patient was instructed to gently rinsing with 0.2% chlorhexidine digluconate twice a day for one week.

**Figure 4:**
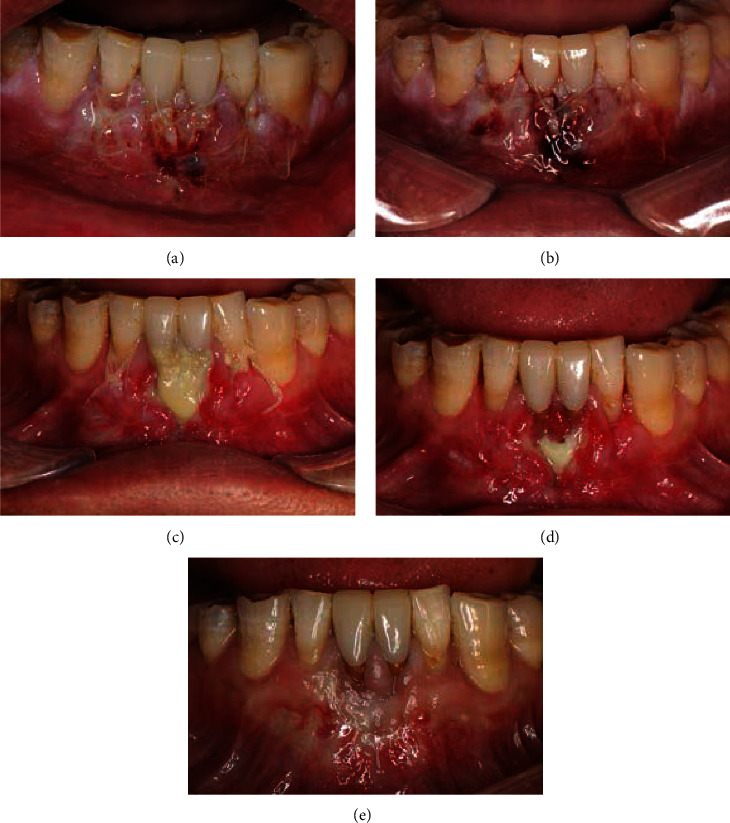
The follow-up intraoral view showed the healing process of the recipient site after (a) 1 day, (b) 2 days, (c, d) 15 days, and (e) 9 months.

The first 2-day follow-up displayed an early wound healing with minimal edema and no infection for the recipient site (Figures 4(a) and 4(b)). At day 15 after surgery, the exposed part of the graft showed ischemic necrosis with a yellowish color (Figure 4(c)). After removing the suture and the superficial necrotic tissue, it was found that the deeper layer of the graft achieved an ideal healing (Figure 4(d)). The 9-month follow-up showed a satisfactory, though not complete, root coverage (residual recession depths of 2, 2, 3, and 2 mm for the four incisors from left to right), excellent chromatic and texture tissue integration of the graft, correction of the scar deformity, great gain in keratinized tissue width and thickness, and deepening of the vestibule.

**Figure 5:**
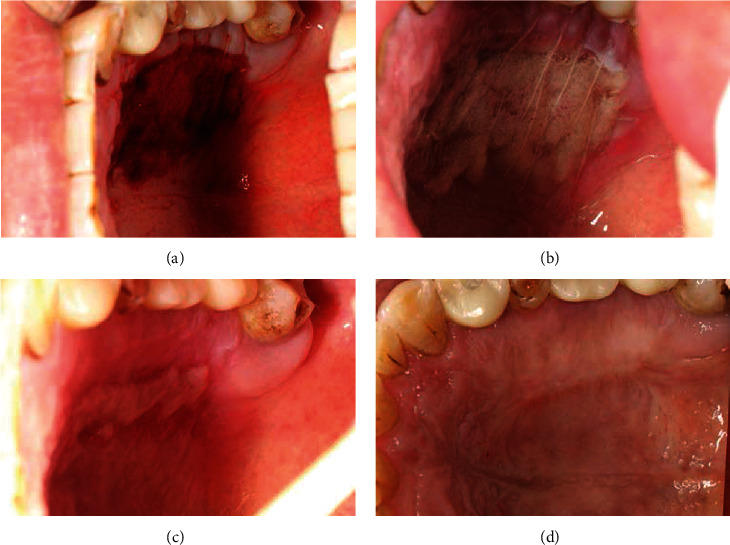
(a) The donor site immediately after surgery. The follow-up intraoral view showed the healing process of the donor site after (b) 2 days, (c) 15 days, and (d) 9 months.

For the donor site, immediately after the surgery, a blood clot was formed on the palatal wound with the help of the collagen matrix (Figure 5(a)). After two days, there was minimal edema and no sign of infection, and a yellowish pseudomembrane was observed (Figure 5(b)). The patient reported of minimal pain, and no pain killer was taken. After 15 days of healing, the wound achieved a near complete closure (Figure 5(c)). The 9-month follow-up showed that the donor site healed well with minimal scar, and no discomfort was reported by the patient (Figure 5(d)).

## 3. Discussion

Treatment of deep GRSF constitutes a clinical challenge owing to several anatomical factors, such as generally large amount of soft tissue missing, scar formation, poor soft tissue quality with poor vascularization, insufficient keratinized tissue, and limited vestibular depth. Hitherto, the report on the treatment of such GRSF is scarce. The present report shows that the large DGG associated with tunnel flap can serve as a one-step procedure for treating GRSF to achieve predicable root coverage, ideal keratinized tissue gain, improvement in soft tissue quality and esthetics, and vestibular depth deepening concurrently.

GRSF is generally associated with inadequate keratinized tissue, and gain of keratinized tissue width is considered to be essential for a long-term success. Traditionally, the gain of keratinized tissue width is believed to be generated by the unsubmerged application of soft tissue graft, exemplified by FGG. The submerged CTG technique associated with pedicle flaps has been thought to generate no or minimal effect of keratinized tissue width gain for a long time. Hence, for GRSF, the FGG procedure may be a preference of many surgeons. Nonetheless, FGG will result in obviously less than ideal results in terms of root coverage and unsatisfactory esthetics of a patch-like appearance. Since the patients are concerned about their esthetic appearance more and more, FGG shall not be the treatment of choice. Laterally positioned flap procedure with CTG was also proposed for root coverage [19]. However, this technique is more suitable for limited (one or two) gingival recessions.

The CTG techniques with CAF or the tunnel flap [18] have been demonstrated to produce high predictability of root coverage [20] and ideal esthetics regarding color and texture match. Delightfully, more and more evidence suggest that they are also effective in gaining keratinized tissue width and thickness [21, 22]. In the present case, DGG instead of subepithelial CTG was used. Compared to the subepithelial CTG, DGG possesses many advantages in terms of obviating the complication of the dehiscence or the necrosis of the primary flap [10], easing to obtain adequate thickness and size of connective tissue graft and, meanwhile, a residual thickness of soft tissue covering the palatal bone. In the present case, a large (30 × 10 mm^2^) DGG was used considering that coronal advancement of the tunnel flap to fully cover the graft is impractical, and the exposed part of graft will have quite a poor early blood supply. According to the study of Yotnuengnit et al., the graft tissue area: visible denuded area ratio is the most important factor to be considered to maximize root coverage when part of the graft on the denuded area is exposed. The size of the graft tissue is suggested to be at least 11 times greater than the visible denuded area [23]. That is why a, as large as possible, DGG was used in our case. In addition, the DGG used was thick (2 mm in thickness) considering that the poor vascularization of the scar tissue in GRSF may lead to considerable shrinkage of the graft. A thicker DGG may lead to obviously thickened keratinized tissue, which may give rise to improved root coverage in the long-term follow-up due to creeping attachment [24]. Additionally, some unexplored variables can have a significant influence on oral environment. The use of probiotics [25] and natural compounds [26] can modify clinical and microbiological parameters in periodontal patients, and they could have an effect also in tissue healing. All these variables should be considered in future clinical trials.

There was a concern of vestibular depth reduction for the tunnel technique. With such a concern, FGG might be the treatment choice of many periodontists for GRSF. Nonetheless, the follow-up of our present case demonstrates that the DGG associated with tunnel technique leads to even deepened vestibular depth. The recipient tunnel bed preparation can displace the muscle attachments followed by the insertion and attachment of the graft to the bony bed precluding their reattachment, and thus, the vestibule is maintained or displaced apically [27]. Superior to FGG, the tunnel technique associated with DGG technique results in a much more natural and esthetic vestibular form.

The surgical technique applied in the case is not a complete tunnel. Due to the unexpected tearing off of the papilla between the two middle incisors at the apical part, modification of leaving the interdental papilla area for secondary healing was made. It would have been possible if the part of the graft left for secondary healing had not been deepithelized. The crowns show high marginal adaptation, which should have no impact on the outcome. One limitation of the present report is short period follow-up period with only one case included. Case series as well as clinical trials of long period follow-up are needed to show the treatment effect. Checklist of the various procedures described in the present report is shown in Table 1.

## 4. Conclusion

The full-split coronally advanced tunnel flap associated with large size DGG leads to predicable root coverage accompanied with ideal keratinized tissue gain, scar correction, and satisfactory esthetics for GRSF, which may constitute a versatile approach for the treatment of GRSF. Further investigation on the clinical effects and predictability of the procedure is required.

## Figures and Tables

**Table 1:** Checklist of the various procedures described in the present report.

Large size DGG associated with tunnel flap
Pedicle flap procedures
Soft tissue/substitute grafting techniques
CTG associated with CAF
CTG associated with tunnel flap
DGG harvesting procedures
Subepithelial CTG harvesting procedures
Laterally positioned flap procedure with CTG
FGG procedure
